# Effect of High Dietary Iron on Fat Deposition and Gut Microbiota in Chickens

**DOI:** 10.3390/ani14152254

**Published:** 2024-08-03

**Authors:** Ting Yang, Shihao Chen, Lingling Qiu, Qixin Guo, Zhixiu Wang, Yong Jiang, Hao Bai, Yulin Bi, Guobin Chang

**Affiliations:** 1 College of Animal Science and Technology, Yangzhou University, Yangzhou 225009, China; 2 Key Laboratory for Animal Genetics & Molecular Breeding of Jiangsu Province, Yangzhou University, Yangzhou 225009, China; 3 Institutes of Agricultural Science and Technology Development, Yangzhou University, Yangzhou 225009, China; 4 Joint International Research Laboratory of Agriculture and Agri-Product Safety of Ministry of Education of China, Yangzhou University, Yangzhou 225009, China

**Keywords:** high dietary iron, poultry fat, 16S rDNA, gut microbiota imbalance

## Abstract

**Simple Summary:**

The rapid growth of the poultry industry has been accompanied by several challenges, of which excessive fat deposition is a major disadvantage. In this study, we found that a high dietary iron (500 mg/kg) intake reduced fat deposition and could reverse the imbalance of gut microbiota induced by a high-fat diet. These findings revealed the role of iron in regulating fat deposition and the gut microbiota of silky fowl black-bone chickens. Our study suggests that iron may regulate fat deposition by influencing the gut microbiota of chickens and provides a potential avenue to prevent excessive fat deposition in chickens by adding iron to the diet.

**Abstract:**

To meet the demand of consumers for chicken products, poultry breeders have made improvements to chickens. However, this has led to a new problem in the modern poultry industry, namely excessive fat deposition. This study aims to understand the effects of dietary iron supplementation on fat deposition and gut microbiota in chickens. In this study, we investigated the effects of iron on the growth performance, fat deposition, and gut microbiota of silky fowl black-bone chickens. A total of 75 7-week-old silky fowl black-bone chickens were randomly divided into three groups (five replicates per group, five chickens per replicate) and fed them for 28 days using a growing diet (control group), a growing diet + 10% tallow (high-fat diet group, HFD group), and a growing diet + 10% tallow + 500 mg/kg iron (HFDFe500 group), respectively. We detected the effects of iron on the growth performance, fat deposition, and gut microbiota of silky fowl black-bone chickens using the growth performance index test, oil red O staining, and HE staining, and found that the high-fat diet significantly increased liver and serum fat deposition and liver injury, while the addition of iron to the diet could reduce the fat deposition caused by the high-fat diet and alleviate liver injury. In addition, 16S rDNA sequencing was used to compare the relative abundance of gut microbiota in the cecal contents in different feeding groups. The results showed that the high-fat diet could induce gut microbiota imbalance in chickens, while the high-iron diet reversed the gut microbiota imbalance. PICRUSt functional prediction analysis showed that dietary iron supplementation affected amino acid metabolism, energy metabolism, cofactors, and vitamin metabolism pathways. In addition, correlation analysis showed that TG was significantly associated with *Firmicutes* and *Actinobacteriota* (*p* < 0.05). Overall, these results revealed high dietary iron (500 mg/kg) could reduce fat deposition and affect the gut microbiota of silky fowl black-bone chickens, suggesting that iron may regulate fat deposition by influencing the gut microbiota of chickens and provides a potential avenue that prevents excessive fat deposition in chickens by adding iron to the diet.

## 1. Introduction

As the world population continues to grow, there is an increasing demand for human consumption of poultry, and poultry products have become a major source of high-quality animal protein [1,2]. Compared with other livestock, poultry not only has a fast growth rate and short generation intervals, but poultry meat and eggs also provide high-quality amino acids and high levels of trace elements [3]. At the same time, poultry also has the advantage of having low initial farming costs due to its small individual scale, and these advantages have led to the rapid development of the poultry industry [2]. Chicken meat is highly favored by consumers because of its high protein content, rich nutrition, and unique flavor. To meet consumer demand for chicken meat, breeders have genetically improved broiler chickens to increase their body weight gain, growth rate, and breast muscle weight significantly [4]. However, the rapid development of the poultry industry is accompanied by many challenges. Excessive fat deposition is an important disadvantage in the poultry industry and can lead to an increase in farm production costs and reduce feed conversion efficiency and product quality. Reducing fat deposition in economically important animals such as chickens can be achieved through different strategies, including genetic selection, feeding strategies, housing and environmental strategies, and hormone supplementation [5]. However, these methods have some problems, such as genetic selection may lead to a decrease in genetic diversity and increase the risk of disease; feeding strategies with excess or insufficient nutrition may result in stunted growth or metabolic disorders in animals.

Fe is an important metallic element that plays a crucial role in biochemical reactions in most organisms [6]. Studies have shown that Fe levels in diets are positively correlated with nitric oxide synthase (NOS) levels in the hypothalamus of animals, which in turn can influence nitric oxide production and regulate appetite, suggesting that Fe may be associated with appetite regulation in animals [7]. It has been reported that dietary Fe levels affect lipid metabolism in animals [8,9], and high levels of dietary Fe can reduce hepatic fatty acid synthesis by decreasing the activity of fatty acid synthesis-related enzymes and the expression of fatty acid synthesis-related genes in the liver of broilers. These changes are speculated to be a factor in the reduction of abdominal fat deposition in broilers [10]. Despite this, most previous studies focus on the changes in iron levels during fat deposition, and few studies have been reported to study fat deposition in chickens with the addition of extra iron to the diet.

The gut in animals is enriched with microorganisms that regulate fat metabolism [11,12], energy balance, and central appetite signaling in the host [13]. It has been shown that the ratio of the phylum Firmicutes to the phylum Bacteroidetes is significantly altered in the gut microbiota of obese humans and mice [14]. Another study found that the transplantation of fecal microbiota from obese mice to recipient germ-free mice resulted in a similar phenotype in the recipient mice [15]. In studies on broiler fat, 12 strains of Lactobacillus were found to reduce triglycerides, abdominal fat deposition, and serum total cholesterol in broilers [16,17]. Additionally, studies have shown that the gut microbiota of chickens plays a key role in fat deposition, with Bacteroides and Lactobacillus being linked to increased body weight gain, abdominal fat deposition, and increased pectoral muscle production [18,19]. Alterations in the gut microbiota can lead to the development of host diseases such as obesity, diabetes [20], and depression [21]. However, it is not yet known whether it is possible to reduce fat deposition in chickens by improving their gut microbiota.

In this study, we investigate the effects of high dietary iron on the growth performance and gut microbiota of silky fowl black-bone chickens under the same feeding environment to understand the effects of high levels of iron on the growth performance, fat deposition, and gut microbiota of the chickens. We also aimed to analyze whether it is possible to regulate the cecum microbiota of the chickens through the addition of Fe in the diets to affect the growth performance and fat deposition in the chickens.

## 2. Materials and Methods

### 2.1. Animals and Experimental Design

In this study, we selected a total of 75 7-week-old silky fowl black-bone chickens with healthy growth and similar weight, purchased from Jiangsu Lihua Animal Husbandry Co., Ltd. (Changzhou, China). All chickens were randomly divided into 3 treatment groups with 5 replicates in each treatment group and 5 chickens in each replicate. The test period was 28 days, with free access to water and feed. The three treatment groups were the control group (growing stage feed), the high-fat diet group (the HFD group, growing stage feed + 10% beef tallow), and the high-fat diet + high iron group (the HFDFe500 group, growing stage diet + 10% beef tallow + 500 mg/kg Fe). The growing stage feed was purchased from Jiangsu Lihua Animal Husbandry Co., Ltd. The feed ingredients of the growing stage feed are shown in Table 1. The supplemental iron was ferric sulfate heptahydrate (reagent grade purity 99.5%) purchased from BBI Life Sciences Corporation (Shanghai, China).

### 2.2. Sample Collection

The chickens were weighed on days 1 and 28 of the experiment to calculate the average daily weight gain (ADG) from days 1 to 28. On the 28th day, all chickens were fasted for 12 h with a normal water supply. On the next morning, 10 chickens were randomly selected from each group and 5 mL of blood was collected from each chicken through the wing vein for serum preparation. The serum was stored at −80 °C for subsequent determination of serum biochemical parameters. After blood collection, the chickens were slaughtered. The liver and abdominal fat were collected and weighed. The liver and abdominal adipose index (tissue weight/BW) were calculated separately and expressed as g/1000 g BW. The collected liver and abdominal fat were stored in a 4% neutral paraformaldehyde solution (Solarbio, Beijing, China) for subsequent histological and morphological observations. Additionally, the contents of the cecum of each chicken were quickly frozen in liquid nitrogen and stored at −80 °C for subsequent DNA extraction.

### 2.3. Determination of Serum Biochemical Parameters

The collected blood was centrifuged at 3500 r/min for 10 min to separate the serum and stored at −20 °C. Total cholesterol (TC) and triglycerides (TG) in serum were determined using an enzyme colorimetric assay kit (Zhongsheng Beizhong Biotechnology Co., Ltd., Beijing, China). Serum aspartate aminotransferase (AST) and alanine aminotransferase (ALT) were determined using a continuous monitoring kit (Zhongsheng Beizhong Biotechnology Co., Ltd., Beijing, China).

### 2.4. Oil Red O Staining

Liver tissue preserved in a 4% neutral paraformaldehyde solution was first embedded, and frozen sections were prepared. The frozen sections were fixed in 10% formalin (Solarbio, Beijing, China) at room temperature, washed with tap water, dried, and then immersed in oil red O staining solution (Solarbio, Beijing, China) while avoiding light. After staining, the sections were washed twice in 60% ethanol (Sinopharm, Shanghai, China) to remove the staining solution and then washed three times in purified water. Immediately thereafter, the sections were re-stained by immersion in hematoxylin staining solution (Solarbio, Beijing, China), washed three times with pure water, immersed in 1% hydrochloric acid differentiation solution (Beyotime, Shanghai, China), and washed twice with purified water. Finally, the stained sections were photographed with each section randomly selected from 3 fields of view.

### 2.5. HE Staining

Abdominal adipose tissue preserved in 4% neutral paraformaldehyde solution was first washed with water. Then, a graded concentration of ethanol was used as a dehydrating agent to remove water from the adipose tissue blocks. The adipose tissue was dehydrated and placed in an immersion bath to make it transparent, and the alcohol in the adipose tissue blocks was replaced. After dehydration and transparency, the adipose tissue blocks were placed in melted paraffin wax for embedding and sectioning. The prepared adipose tissue sections were deparaffinized in xylene (Sinopharm, Shanghai, China), and then washed with a gradient concentration of ethanol and distilled water to remove the xylene and bring the sections into water. The treated adipose tissue sections were stained in hematoxylin solution, washed with distilled water after staining, and decolorized in 1% hydrochloric acid–ethanol solution (hydrochloric acid: 75% ethanol = 1:100, Sinopharm, Shanghai, China). The sections were soaked in 1% ammonia (Sinopharm, Shanghai, China) to restore the blue color, stained with eosin ethanol solution (Solarbio, Beijing, China), washed with distilled water, dehydrated with ethanol, and made transparent with xylene. The stained sections were sealed with neutral resin (Solarbio, Beijing, China) and photographed under a microscope with 3 randomly selected fields of view for each section.

### 2.6. DNA Extraction, Library Preparation, and 16S rDNA Gene Sequence

DNA from different samples was extracted using CTAB (cetyltrimethylammonium bromide) according to the manufacturer’s instructions [22]. The reagent, which was designed to recover DNA from trace amounts of sample, is effective for the preparation of DNA of most bacteria. Nuclear-free water was used for blanks. The total DNA was eluted in 50 μL of Elution buffer and stored at −80 °C until measurement in the PCR by LC-Bio Technology Co., Ltd. (Hangzhou, China). Following that, the universal primers 341F (5′-CCTACGGGNGGCWGCAG-3′) and 805R (5′-GACTACHVGGGTATCTAATCC-3′) were used to amplify the V3–V4 hypervariable region of the 16S rDNA gene. The 5′ ends of the primers were tagged with specific barcodes per sample and sequencing universal primers. PCR amplification was performed in a total volume of 25 μL reaction mixture containing 25 ng of template DNA, 12.5 μL PCR Premix, 2.5 μL of each primer, and PCR-grade water to adjust the volume. The PCR conditions to amplify the prokaryotic 16S fragments consisted of an initial denaturation at 98 °C for 30 s; 32 cycles of denaturation at 98 °C for 10 s, annealing at 54 °C for 30 s, and extension at 72 °C for 45 s; and then final extension at 72 °C for 10 min. The PCR products were confirmed with 2% agarose gel electrophoresis. Throughout the DNA extraction process, ultrapure water, instead of a sample solution, was used to exclude the possibility of false-positive PCR results as a negative control. The PCR products were purified by AMPure XT beads (Beckman Coulter Genomics, Danvers, MA, USA) and quantified by Qubit (Invitrogen, Carlsbad, CA, USA). The amplicon pools were prepared for sequencing and the size and quantity of the amplicon library were assessed on Agilent 2100 Bioanalyzer (Agilent, Santa Clara, CA, USA) and with the Library Quantification Kit for Illumina (Kapa Biosciences, Woburn, MA, USA), respectively. The libraries were sequenced on the NovaSeq PE250 platform (Illumina, San Diego, CA, USA).

### 2.7. Analysis of 16S rDNA Gene Sequence

Samples were sequenced on the Illumina NovaSeq platform, according to the manufacturer’s recommendations, provided by LC-Bio Technology Co., Ltd., Hangzhou, China. Paired-end reads were assigned to samples based on their unique barcode and truncated by cutting off the barcode and primer sequence. Paired-end reads were merged using FLASH. Quality filtering on the raw reads was performed under specific filtering conditions to obtain high-quality clean tags according to fqtrim (v0.94). Chimeric sequences were filtered using Vsearch software (v2.3.4). After dereplication using DADA2, we obtained the ASV feature sequence and an Amplicon Sequence Variant (ASV) feature table. The alpha diversity analysis and beta diversity analysis were conducted based on the ASV feature sequence and ASV feature abundance table. Alpha diversity is applied in analyzing the complexity of species for a sample through Chao1 and Shannon, and all these indices in our samples were calculated with QIIME2 (2019.7). Beta diversity is applied in analyzing species diversity among different environment communities through PCoA and PLS-DA. Principal Coordinates Analysis (PCoA) is based on a distance matrix, which is used to rearrange samples in a visualized low-dimensional space to maximize the display of the relationship between samples. The closer the distance between the sample points, the more similar the species composition structure between the samples. Partial Least Squares Discrimination Analysis (PLS-DA) is a supervised discriminant analysis of variance method to maximize the differences between groups, which uses partial least squares regression to model the relationship between metabolite expression and sample category to achieve modeling prediction of samples. In general, both values of R2 and Q2 should be >0.5. Then, the ASV feature sequences were annotated with the SILVA database and the abundance of each species in each sample was analyzed according to the ASV feature abundance table. The top 10 microbial taxa in relative abundance were visualized at the phylum and genus levels. The linear discriminant analysis effect size (LEfSe) analysis algorithm with a linear discriminant analysis (LDA) score of 3.0 was used to identify the gut microbiota with statistical significance at different taxonomic levels [23]. PICRUSt2 functional analysis (https://github.com/picrust/picrust2, accessed on 20 May 2024) based on the COG database was used to annotate the function of the differential abundance of gut microbiota.

### 2.8. Statistical Analysis

All data were statistically analyzed using SPSS 25.0 (SPSS Inc., Chicago, IL, USA). A one-way ANOVA was used for multiple-group comparison analysis. The Kruskal–Wallis test was used for gut microbiota. Duncan’s multiple range test was used to determine the significance. All data are expressed as mean ± SEM. *p* < 0.05 indicates significant difference; *p* < 0.01 indicates highly significant difference.

## 3. Results

### 3.1. Growth Performance

After feeding silky fowl black-bone chickens with different diets for 28 days, we slaughtered these chickens and measured the ADG, liver yield, and abdominal adipose tissue yield. We found that the high-fat diet significantly (*p* < 0.05) increased the ADG from day 1 to 28 compared with the control group. The addition of high Fe to the high-fat diet significantly decreased the ADG from day 1 to 28 compared with the HFD group (*p* < 0.05) (Table 2). Additionally, the high-fat diet significantly (*p* < 0.05) increased liver yield and abdominal adipose tissue yield compared to the control group (Table 2). Similarly, the addition of high Fe to the high-fat diet significantly (*p* < 0.05) decreased liver yield and abdominal adipose tissue yield compared to the HFD group (Table 2). These results suggest that the addition of high Fe to the diet may reduce fat deposition in chickens.

### 3.2. Serum Biochemical Parameters

The results of serum biochemical parameters tests showed that high-fat diets significantly increased the concentrations of serum total cholesterol (TC) and serum triglycerides (TG) (*p* < 0.05), and the addition of high Fe to high-fat diets significantly decreased the concentrations of TC and TG (*p* < 0.05) (Table 3). Serum aminotransferase assays showed that ALT levels significantly increased (*p* < 0.05) with the addition of high fat to the basal diet, and significantly decreased (*p* < 0.05) with the addition of high iron to the high-fat diet, with a significant difference between the three groups (*p* < 0.05) (Table 3). AST levels significantly increased (*p* < 0.05) with the addition of high fat to the basal diet. However, AST levels only decreased by 18.04% (*p* > 0.05) after the addition of high Fe to high-fat diets (Table 3). These results indicate that the addition of high Fe to the diet could reduce the fat deposition in silky fowl black-bone chickens and help maintain the normal physiological function of the liver.

### 3.3. Lipid Deposition and Adipose Tissue Development

**Figure 1:**
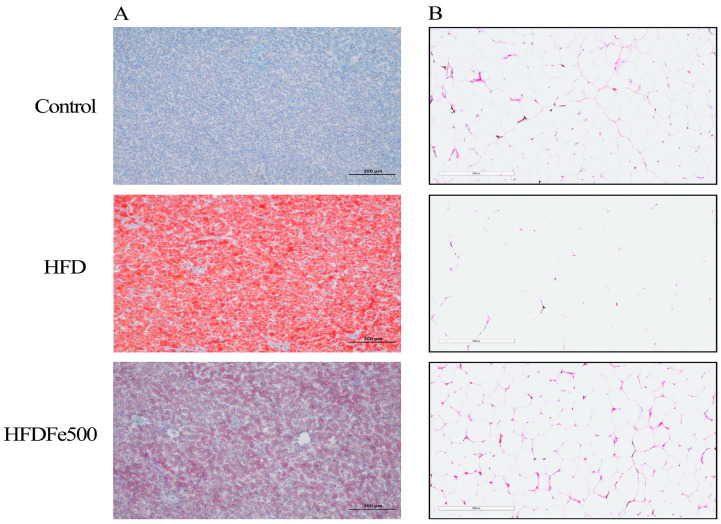
Effects of high iron diet on lipid metabolism in the different tissues. (**A**) Histological analysis of liver sections with oil red O staining. (**B**) Histological analysis of abdominal adipose tissue sections with hematoxylin and eosin staining (scale bar = 20 μm).

To understand the effect of Fe addition on fat deposition, we performed oil red O staining and HE staining experiments, respectively. Oil red O staining results showed that the number of red lipid droplets in the liver tissue of the HFD group was significantly increased compared to the control group, while the number of red lipid droplets in the liver tissue of the HFDFe500 group was significantly decreased compared to the HFD group (Figure 1A). HE staining of abdominal fat showed that fat vacuoles in the HFD group were larger and the number of fat vacuoles per unit area decreased compared to the control group. In contrast, fat vacuoles in the HFDFe500 group were smaller and the number of fat vacuoles per unit area increased compared to the HFD group (Figure 1B). These results indicate that dietary Fe supplementation can alleviate fat deposition.

### 3.4. Alpha Diversity and Beta Diversity Analysis of Gut Microbiota

**Figure 2:**
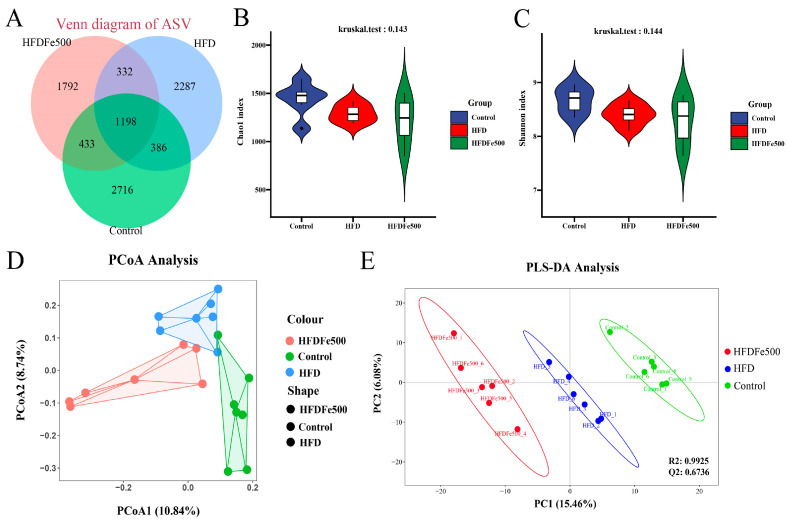
Analysis of the amplicon sequence variant and α diversity of the gut microbiota in chickens. (**A**) Venny plots of amplicon sequence variant (ASV) in the cecum of chickens in the three groups. (**B**) α-diversity analyses based on the Chao1 index. (**C**) α-diversity analyses based on the Shannon index. (**D**) β-diversity analyses based on the PCoA plot about the cecal microbiota. (**E**) β-diversity analyses based on the PLS-DA sample plot with confidence ellipse plots.

To understand whether increasing dietary Fe content can affect the cecal microbiota of silky fowl black-bone chickens, we analyzed the cecal microbiota of the HFDFe500, HFD, and control groups using 16S rDNA amplification sequencing. Taxonomy results of all bacteria are shown in Supplementary Tables S1 and S2. In total, 28 phyla and over 400 genera were identified after data filtering. All results were subsequently used for further differential and functional analyses. The Venn diagram illustrates the number of ASVs common and unique to each group. A total of 1198 ASVs were common to the three groups, whereas 1792, 2287, and 2716 ASVs were unique to the HFDFe500, HFD, and control groups, respectively (Figure 2A). Alpha diversity analysis indicated that the Chao1 index (Kruskal–Wallis test *p* > 0.05, Figure 2B) and the Chao1 index (Kruskal–Wallis test *p* > 0.05, Figure 2C) showed no significant difference in species diversity of cecal microbiota among the three groups. These findings suggest that the effects of different diets on the cecal microbial communities of silky fowl black-bone chickens are mainly due to changes in bacterial community composition.

In addition, β-variation was calculated to assess changes in the cecal microbiota community. PCoA analysis showed significant differences between the HFD group and the other two groups (the control group and the HFDFe500 group) (Figure 2D). The supervised analysis of PLS-DA focuses on the partitioning of the three groups, showing an R2 value of 0.9925 and a Q2 value of 0.6736, suggesting that the current PLS-DA model is reliable and the three groups are partitioned (Figure 2E).

### 3.5. Analysis of Gut Microbiota Community Structure

**Figure 3:**
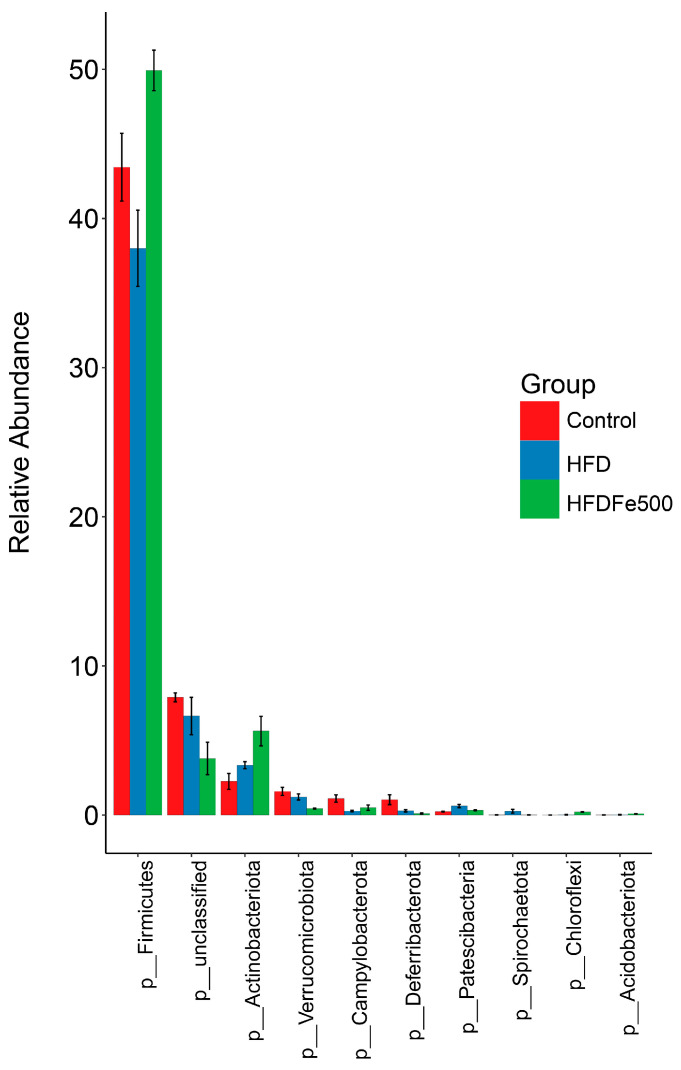
The differences between the cecal microbiota of different groups at the phylum level.

**Figure 4:**
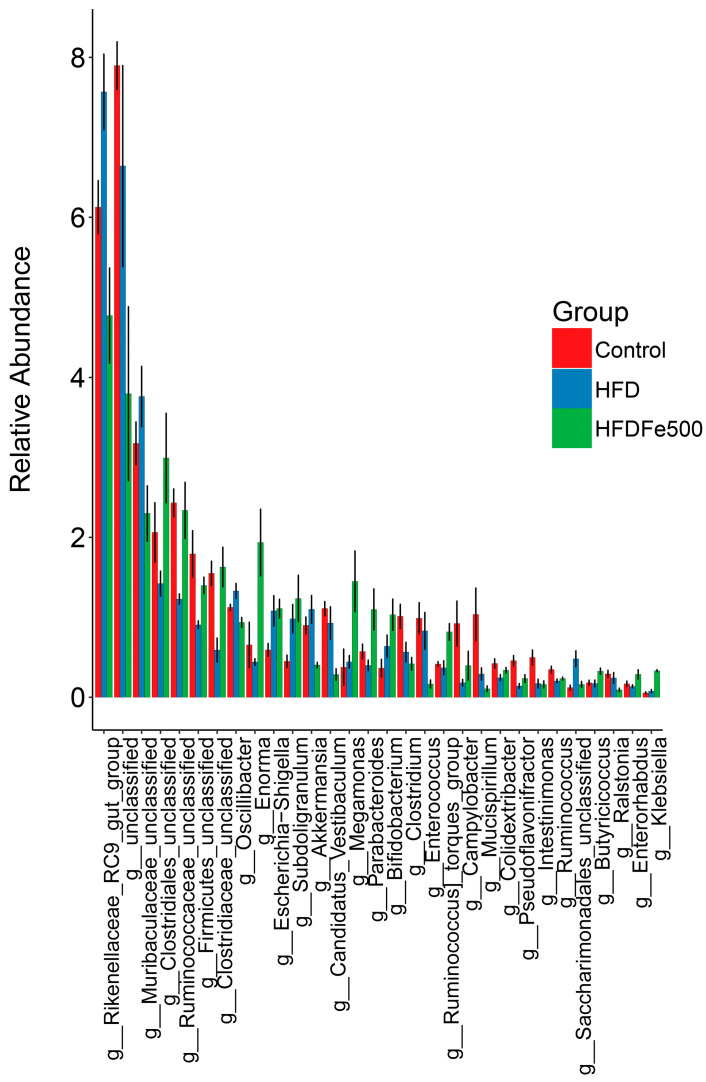
The differences between the cecal microbiota of different groups at the genus level.

**Figure 5:**
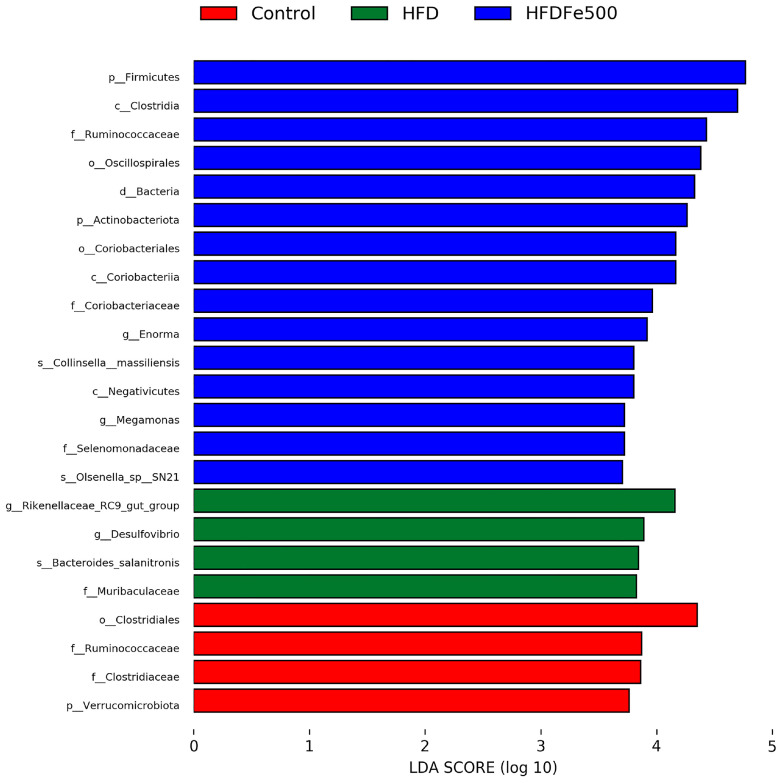
Biomarkers of discriminative bacteria (from phylum to genus) in different groups identified by LEfSe analysis (LDA score > 3.7). The length of the bars in the chart represents the influence sizes of the different species (i.e., LDA score).

To further clarify the effects of high-iron and high-fat diets on gut microbiota composition, we analyzed the relative abundance of microbial taxa. We examined the 10 bacteria with the highest relative abundance at the phylum level and genus level (Table 4 and Table 5). *Firmicutes* and *Bacteroidota* were the two most abundant phyla. Different diets led to changes in the relative abundance of these bacteria. Compared to the control group, the high-fat diet decreased the relative abundance of *Firmicutes* and *Campylobacterota* while increasing the abundance of *Bacteroidota*, *Desulfobacterota*, and *Synergistota*. Conversely, the high-iron diet restored their relative abundance. The *Firmicutes/Bacteroidetes* ratio was significantly lower in the HFD group compared to the control group and was restored with the addition of iron (*p* < 0.05). Differential analysis of bacterial communities at the genus level showed that *Bacteroides*, *Rikenellaceae_RC9_gut_group*, *Prevotellaceae_UCG-001*, *Faecalibacterium*, and *Desulfovibrio* were the top five abundant bacterial communities, constituting 85% of the total bacterial biomass. The most abundant genus in the control group and HFDFe500 group was *Bacteroides*, while the most abundant genus in the HFD group was *Prevotellaceae_UCG-001*. Then, we also analyzed the differences between the cecal microbiota of different groups at the phylum and genus levels. As shown in Figure 3, there were nine different microbiota at the phylum level, including *Firmicutes* and *Campylobacterota*. As shown in Figure 4, there were 29 different microbiota at the genus level, such as *Rikenellaceae_RC9_gut_group*. In addition, we further used LEfSe to analyze the differences between the cecum microbiota of different groups and to understand the effect of different diets on cecum microbiota. LEfSe analysis identified 23 ASVs as biomarkers at a threshold of LDA score > 3.7 (Figure 5). Analysis at the phylum and genus level showed only one biomarker bacterium in the control group (*Verrucomicrobiota*), two biomarker bacteria in the HFD group (*Rikenellaceae_RC9_gut_group* and *Desulfovibrio*), and four biomarker bacteria in the HFDFe500 group (*Firmicutes*, *Actinobacteriota*, *Enorma*, and *Megamonas*).

### 3.6. Functional Prediction of the Differential Gut Microbiota

**Figure 6:**
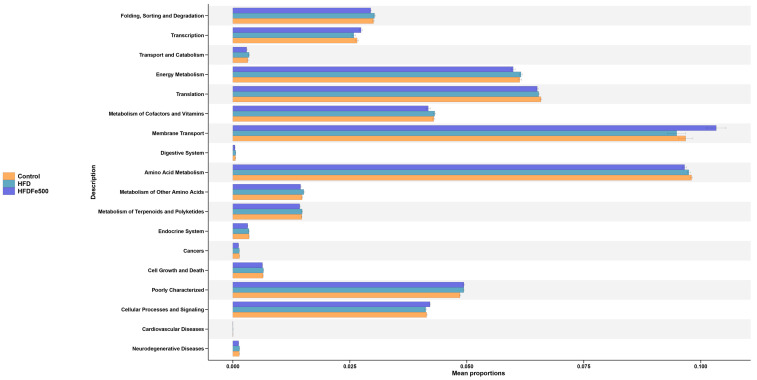
PICRUSt2 functional prediction analysis of the differential abundant bacterial communities between different groups.

For functional prediction analysis, we performed PICRUSt2 functional prediction of the gut microbiota (Figure 6, Supplementary Tables S3 and S4). The differential KEGG pathway mainly focuses on folding, sorting, and degradation; transcription; energy metabolism; translation; metabolism of cofactors and vitamins; membrane transport; amino acid metabolism; poorly characterized; and cellular processes and signaling. Among them, metabolic processes are mainly concentrated in energy metabolism, metabolism of cofactors and vitamins, and amino acid metabolism, which are closely related to growth and fat generation.

### 3.7. Relationship between the Gut Microbiota with ADG, Liver Yield, and Abdominal Adipose Tissue Yield

**Figure 7:**
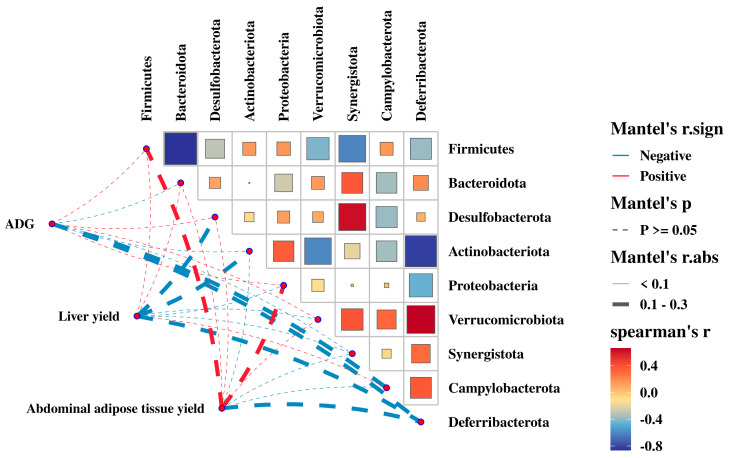
The correlation analysis of the growth performance and the relative abundances of cecal bacteria (phyla level).

To understand the effect of gut microbiota on growth performance, we analyzed the relationship between gut microbiota and growth performance indicators, as well as the interaction of gut microbiota in regulating growth performance at the phylum level (Figure 7, Supplementary Table S5). There was no significant correlation between bacterial phylum and growth performance (*p* > 0.05); however, it can be seen that *Firmicutes* were negatively correlated with *Synergistota* and *Deferribacterota*, and synergized with *Proteobacteria* to regulate ADG. *Firmicutes* were negatively correlated with *Desulfobacterota* and *Verrucomicrobiota*, and *Synergistota* and *Deferribacterota* negatively correlated with *Campylobacterota* and synergized with *Campylobacterota* to regulate liver yield. *Firmicutes* were negatively correlated with *Synergistota* and *Deferribacterota* and synergized with *Proteobacteria* to regulate abdominal adipose tissue yield. The bacteria regulated each other and collectively affected growth performance, although these effects were not significant in this study.

### 3.8. Relationship between the Gut Microbiota with Serum Biochemical Parameters

**Figure 8:**
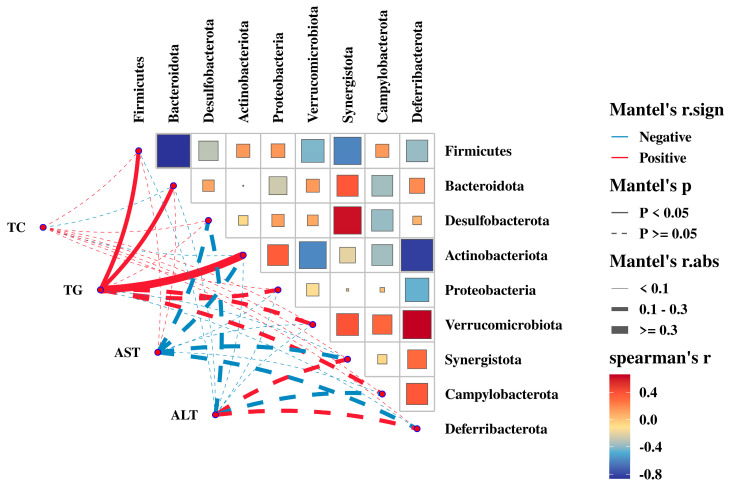
The correlation analysis of the serum biochemical parameters and the relative abundances of cecal bacteria (phyla level).

We also analyzed the relationship between gut microbiota and serum biochemical parameters, as well as the interaction of bacteria in regulating serum biochemical parameters at the phylum level (Figure 8, Supplementary Table S6). The correlation between TG and *Firmicutes* and *Actinobacteriota* was significant (*p* < 0.05), and *Firmicutes* and *Actinobacteriota* synergistically regulated TG. Other than that, there was no significant correlation between *Bacteroidota* and TG (*p* > 0.05), and no significant correlation between *Bacteroidota* and TC, AST, and ALT (*p* > 0.05). Specifically, *Firmicutes* were positively correlated with *Proteobacteria* and *Campylobacterota*, and synergistically regulated TC. *Firmicutes* were positively correlated with *Desulfobacterota*, *Verrucomicrobiota*, and *Synergistota*, and *Deferribacterota* negatively correlated with *Desulfobacterota*, *Verrucomicrobiota*, and *Synergistota*, and antagonistically regulated AST. *Firmicutes* negatively correlated with *Desulfobacterota* and *Verrucomicrobiota*, and antagonistically regulated ALT. The bacteria regulated each other and collectively affected TG, TC, AST, and ALT, further suggesting that gut microbiota regulate fat deposition.

## 4. Discussion

In animal husbandry, the fat output of animals has always been a trait of great interest which affects feed remuneration, disease resistance, reproductive performance, and meat quality [24]. Therefore, the study of fat deposition in chickens is beneficial to understand its influencing factors and providing a theoretical basis for reducing fat deposition in poultry. In this study, we aimed to understand the effects of iron on fat deposition, cecal microbial composition, and function in the chickens.

Fats and oils can promote growth and efficiency in poultry [25,26], and similar phenomena were found in the present study. In the present study, we found that the daily weight gain of silky fowl black-bone chickens increased significantly in the HFD group, and this result suggests that the addition of tallow to the basal diet could promote the growth of silky fowl black-bone chickens. However, overuse of fats and oils may lead to the opposite effect. Bozkurt et al. added supplemental fats at concentrations of 0.6%, 1.2%, and 1.8% to the commercial dietary base for laying hens and found that laying hens in the 1.2% and 1.8% fat concentration groups had significantly higher abdominal fat percentages, appeared to have an over-expanded abdomen, and exhibited depression compared to the group that was supplemented with the 0.6% fat concentration [27]. In the present study, we found similar results, in that both liver and abdominal fat production was significantly increased in the HFD group compared to the control group, and the serum biochemical parameters of TC, TG, AST, and ALT content were also significantly increased. In addition, oil red O staining and HE staining showed that liver fat and abdominal fat increased in the HFD group compared with the control group. These results suggest that high-fat diets not only promote the growth of chickens but also produce the negative effect of increased fat deposition.

Fe is an essential trace element for living organisms, participating in signaling, oxygen transmission, energy metabolism, and other physiological reactions [28]. Numerous studies have shown that fat metabolism in animals is affected by Fe intake levels, and the two are closely related. Choi et al. found that increasing Fe levels in the diet of mice could reduce their high-density lipoprotein cholesterol levels [9]. Don Giovanni et al. found a significant increase in fasting blood glucose and TG levels, and a decrease in visceral adipose tissue weight and mean adipocyte size, by adding excess Fe to mouse diets [29]. These studies suggest that the amount of Fe in the diet can influence fat deposition in animals. In the present study, we found that ADG, liver index, and abdominal fat index were significantly lower in the HFDFe500 group compared with the HFD group, and fat deposition in serum and liver was also significantly lower. Additionally, serum AST and ALT were reduced, and liver injury was alleviated. In conclusion, the addition of 500 mg/kg of Fe to the diet helped reduce fat deposition in silky fowl black-bone chickens. It has been reported that iron regulates mitochondrial fat oxidation and influences adipose tissue thermogenesis. Thermogenesis is a process that increases energy expenditure, and adipose tissue is a tissue that generates heat through mitochondrial fuel oxidation. Iron deficiency may impair mitochondrial fuel oxidation by inhibiting iron-containing molecules, resulting in decreased energy expenditure [30].

The gut microbiota plays a key role in regulating the gut microenvironment and coordinating various physiological aspects of digestion, absorption, and metabolism. Imbalance of gut microbiota homeostasis can lead to dysfunction of various systems, so maintaining gut microbiota homeostasis is crucial in regulating the development of diseases. The study reported that high-fat diets can induce nonalcoholic steatohepatitis and dysbiosis of gut bacteria, with changes in the abundance of the Bacteroides and Lach-nospiraceae genera closely associated with the degree of liver fibrosis and the severity of nonalcoholic steatohepatitis [31]. Our findings suggest that the high-fat diet decreased the relative abundance of *Firmicutes* and *Campylobacterota* while increasing the abundance of *Bacteroidota*, *Desulfobacterota*, and *Synergistota*, whereas Fe reversed the microbiota imbalance induced by the high-fat diet. The dominant bacterial species and their composition ratios vary significantly among different diseases in gut microbiota. In this study, *Firmicutes* and *Bacteroidetes* were the dominant bacterial species, similar to many other studies [32]. Compared to healthy individuals, patients with nonalcoholic fatty liver disease and alcoholic fatty liver disease have a higher proportion of *Bacteroidetes/Firmicutes* [33]. Similar results were also found in this study. A high-fat diet reduces the relative abundance of *Firmicutes* and increases the relative abundance of *Bacteroidetes*, raising the *Bacteroidetes/Firmicutes* ratio, while a high dietary iron intake reverses this change. The results suggest that high-fat diets cause inflammation in the gut, and that iron can alleviate inflammation in the gut. Additionally, an increased ratio of *Bacteroides* mimeticus and *Bacteroides* thicketi leads to dysregulation of the bile acid pool, resulting in increased energy expenditure and a chronic inflammatory state, which further disrupts the gut ecological balance and bile acid biosynthesis [34]. We also found significant changes in *Campylobacterota*. *Campylobacterota*, previously known as *Epsilonproteobacteria*, is a group of predominantly Gram-negative, spiral-moving bacteria. Despite their diverse environments, they share a common mechanism of energy conservation [35]. Gut microbial populations of gilthead seabream (*Sparus aurata*) were altered following the addition of a dietary mixture of bile salts, which regulate lipid metabolism and fat content. In the hindgut, the relative abundance of *Campylobacterota* decreased when bile salts were added, consistent with our findings, where a high-fat diet significantly decreased the relative abundance of *Campylobacterota*, and a high dietary iron intake reversed this change. Moreover, the relative abundance of *Desulfobacterota* also doubled in the foregut, similar to our study, where a high-fat diet increased the relative abundance of *Desulfobacterota*, although this difference was not significant, while high dietary iron decreased the relative abundance of *Desulfobacterota* [36]. The only bacterium that differed significantly at the genus level was *Rikenellaceae_RC9_gut_group*. In Tan sheep, the *Rikenellaceae_RC9_gut_group* was shown to be significantly positively correlated with meat fat [37]. High-fat diets can increase the abundance of *Rikenellaceae* and *Rikenellaceae_RC9_gut_group*, regulating lipid deposition traits by altering abundance [38,39]. A similar phenomenon was found in our study where a high dietary iron intake increased the relative abundance of *Rikenellaceae_RC9_gut_group*, while a high dietary iron intake significantly decreased its relative abundance. All results indicate that a high dietary iron intake reverses the high-fat diet-induced imbalance in the gut microbiota, suggesting that Fe plays a role in maintaining gut microbial homeostasis.

It was found that the activated hypoxia-inducible factor HIF-1α promotes iron uptake and thus maintains mitochondrial function by transcriptionally regulating Tfr1 expression during beige fat formation [40]. Mitochondria are directly related to energy, which is consistent with the results of the present study. PICRUSt2 function prediction results showed that the differential pathway was enriched in energy metabolism, metabolism of cofactors and vitamins, and amino acid metabolism. It has been reported that cofactors and vitamins are essential for proper fat metabolism and that they support enzyme activity, influence energy production, and regulate the function and number of adipocytes through a variety of mechanisms [41]. In addition, amino acid metabolism affects fat deposition in several ways, such as energy supply, protein synthesis, hormone regulation, fatty acid synthesis, and microbiome balance [41]. These results once again prove that this study is reliable.

Bacterial communities can interact synergistically or antagonistically, with bacteria of similar degradation orientations usually acting synergistically, such as *Bifidobacterium* and *Lactococcus* in carbohydrate metabolism [42]. In this study, *Firmicutes* synergized with *Proteobacteria* to regulate ADG and abdominal adipose tissue yield, with *Campylobacterota* to regulate liver yield, and with *Actinobacteriota* to regulate TG. Additionally, *Deferribacterota* antagonistically regulates AST with *Desulfobacterota*, *Verrucomicrobiota*, and *Synergistota*, and *Firmicutes* antagonistically regulates ALT with *Desulfobacterota* and *Verrucomicrobiota*. In summary, the gut microbiota is associated with growth performance and fat metabolism in chickens and is regulated by interactions between gut microbiota.

## 5. Conclusions

In the current study, we found that a high dietary iron (500 mg/kg) intake could reduce fat deposition and alleviate liver injury induced by a high-fat diet. In addition, we also found that a high dietary iron (500 mg/kg) intake could reverse the high-fat diet-induced gut microbiota imbalance and gut microbiota interact to regulate growth performance and fat deposition in chickens. These findings revealed the role of iron in regulating fat deposition and the gut microbiota of silky fowl black-bone chickens. Our study suggests that iron may regulate fat deposition by influencing the gut microbiota of chickens and provides a potential avenue that prevents excessive fat deposition in chickens by adding iron to the diet.

## Figures and Tables

**Table 1:** Compositions and nutritional contents of the experimental diets.

Items	Content
Ingredient (%)	
Corn	62.49
Flour	2.00
Soybean meal	25.20
Corn protein flour	2.00
Soybean oil	1.96
Stone powder	1.24
Calcium hydrogen phosphate	1.11
Choline chloride	1.00
Premix	3.00
Nutritional level (%)	
Crude protein	18.50
Metabolizable energy (MJ/kg)	12.55
Ca	0.80
Available phosphorus	0.33
Digestible lysine	0.90
Iron (mg/kg)	321.00

Premix provided for each kilogram of diet: VA: 7500 IU, VD: 3000 IU, VE: 50 IU, VK3: 50 mg; VB1: 90 mg; VB2: 300 mg; VB6: 60 mg; VB12: 0.4 mg; VB3: 1000 mg; VB5: 300 mg, folate: 20 mg; biotin: 2.0 mg; Fe: 1.3 g; Cu: 0.25 g; Zn: 2.0 g; Mn: 2.35 g; I: 20.0 mg; and Se: 4.5 mg. Except for crude protein, whose value was measured, the levels of all nutrients were calculated 1.

**Table 2:** Effect of different diets on the growth performance.

Items	Control	HFD	HFDFe500	*p* Value
ADG (g)	27.52 ± 5.66 ^b^	35.30 ± 4.96 ^a^	26.63 ± 6.15 ^b^	0.003
Liver yield (g/1000 g BW at slaughter)	19.99 ± 3.17 ^b^	25.88 ± 5.45 ^a^	18.74 ± 1.77 ^b^	0.001
Abdominal adipose tissue yield (g/1000 g BW at slaughter)	23.99 ± 14.97 ^b^	41.07 ± 6.24 ^a^	29.75 ± 10.82 ^b^	0.008

^a,b^ Within a row for each item, different superscripts indicate significant differences (*p* < 0.05). ADG, average daily gain; BW, body weight.

**Table 3:** Effect of different diets on the serum biochemical parameters.

Items	Control	HFD	HFDFe500	*p* Value
TC (mmol/L)	3.78 ± 0.31 ^c^	14.17 ± 1.54 ^a^	9.26 ± 1.92 ^b^	0.001
TG (mmol/L)	1.75 ± 0.18 ^c^	3.87 ± 0.26 ^a^	2.72 ± 0.24 ^b^	<0.001
AST (U/L)	230.59 ± 9.16 ^b^	305.42 ± 15.33 ^a^	250.32 ± 5.22 ^ab^	0.040
ALT (U/L)	2.51 ± 0.27 ^b^	7.66 ± 1.82 ^a^	3.39 ± 0.74 ^b^	0.014

^a,b,c^ Within a row for each item, different superscripts indicate significant differences (*p* < 0.05). TG, triglyceride; TC, total Cholesterol; ALT, alanine aminotransferase; AST, aspartate aminotransferase.

**Table 4:** Effect of different diets on the relative bacterial abundances (%, level of phyla).

Phyla	Control	HFD	HFDFe500	*p* Value
Firmicutes	43.60 ± 2.28 ^b^	38.41 ± 2.54 ^b^	50.41 ± 1.41 ^a^	0.004
Bacteroidota	35.80 ± 2.47 ^ab^	40.85 ± 2.23 ^a^	31.88 ± 1.82 ^b^	0.035
unclassified	7.93 ± 0.30 ^a^	6.72 ± 1.27 ^ab^	3.83 ± 1.10 ^b^	0.028
Desulfobacterota	3.57 ± 0.63	5.27 ± 0.50	3.84 ± 0.58	0.112
Actinobacteriota	2.27 ± 0.53 ^b^	3.39 ± 0.24 ^b^	5.69 ± 1.00 ^a^	0.008
Proteobacteria	2.28 ± 0.17	2.73 ± 0.18	2.80 ± 0.26	0.188
Verrucomicrobiota	1.60 ± 0.28 ^a^	1.23 ± 0.21 ^a^	0.44 ± 0.03 ^b^	0.003
Synergistota	0.79 ± 0.30	0.82 ± 0.10	0.49 ± 0.12	0.434
Campylobacterota	1.12 ± 0.25 ^a^	0.28 ± 0.05 ^b^	0.50 ± 0.18 ^b^	0.013
Deferribacterota	1.04 ± 0.33 ^a^	0.29 ± 0.08 ^b^	0.11 ± 0.04 ^b^	0.011

^a,b^ Within a row for each item, different superscripts indicate significant differences (*p* < 0.05).

**Table 5:** Effect of different diets on the relative bacterial abundances (%, level of genera).

Genera	Control	HFD	HFDFe500	*p* Value
Bacteroides	23.89 ± 5.09	16.18 ± 2.40	26.22 ± 6.55	0.354
Rikenellaceae_RC9_gut_group	18.93 ± 0.84 ^a^	19.53 ± 1.39 ^a^	12.69 ± 1.59 ^b^	0.004
Prevotellaceae_UCG-001	18.05 ± 4.09	21.30 ± 2.27	14.30 ± 4.91	0.467
Faecalibacterium	13.70 ± 1.69	15.03 ± 1.65	21.72 ± 3.85	0.098
Desulfovibrio	10.84 ± 1.97	13.03 ± 1.05	9.66 ± 1.40	0.311
Olsenella	1.79 ± 0.24	3.16 ± 0.41	4.04 ± 1.88	0.383
Ligilactobacillus	3.38 ± 0.43	3.19 ± 0.13	3.05 ± 0.35	0.779
Oscillibacter	3.52 ± 0.27	3.43 ± 0.27	2.49 ± 0.20	0.058
Escherichia-Shigella	1.81 ± 0.19	2.79 ± 0.49	2.92 ± 0.28	0.075
Merdimonas	4.08 ± 0.47	2.36 ± 0.36	2.92 ± 0.96	0.196

^a,b^ Within a row for each item, different superscripts indicate significant differences (*p* < 0.05).

## Data Availability

The 16s rDNA raw sequence data have been submitted to NCBI SRA (PRJNA1121650).

## Supplementary Materials

The following supporting information can be downloaded at: https://www.mdpi.com/article/10.3390/ani14152254/s1, Table S1: Taxonomy results of all bacteria at the phylum level; Table S2: Taxonomy results of all bacteria at the genus level; Table S3: The results of the expression data of the functional genes predicted by PICRUST2; Table S4: The statistical differences of different groups predicted by PICRUST2; Table S5: Relationship between the gut microbiota with ADG, liver yield and abdominal adipose tissue yield; Table S6: Relationship between the gut microbiota with serum biochemical parameters.
